# Study on the Influence of Microstructure on the Rolling Contact Fatigue Performance of Silicon Nitride Ceramics Balls

**DOI:** 10.3390/ma19091892

**Published:** 2026-05-04

**Authors:** Feng Sun, Dechang Jia, Bin Li, Tingxia Dong, Changsheng Shen, Yelei Zhang, Zaiyi Wang, Weiru Zhang

**Affiliations:** 1 Institute for Advanced Ceramics, Harbin Institute of Technology, Harbin 150001, China; 2 Sinoma Advanced Nitride Ceramics Co., Ltd., CNBM Group, Zibo 255000, China; libin@sinomaceramic.com (B.L.); wzy@sinomaceramic.com (Z.W.);

**Keywords:** Si_3_N_4_ ceramic balls, sintering aids, microstructure, rolling contact fatigue (RCF), failure analysis

## Abstract

**Highlights:**

- Al_2_O_3_-Y_2_O_3_ content non-monotonically regulates β-Si_3_N_4_ aspect ratio, peaking at 4.47 at 10 wt% addition.
- Low sintering aid causes porosity and spalling; excessive aid leads to soft grain boundary phase and wear failure.
- 10 wt% Al_2_O_3_- Y_2_O_3_ yields fully dense Si_3_N_4_ balls with max RCF life of 1.035 × 10^7^ cycles, no Si_2_N_2_O phase.

**Abstract:**

This study prepares three types of Si_3_N_4_ ceramic bearing balls with distinct microstructures by regulating the content of Al_2_O_3_-Y_2_O_3_ sintering aids, and systematically investigates the influence mechanisms of microstructure, grain boundary phase distribution and grain aspect ratio on the rolling contact fatigue (RCF) failure behavior. The experimental results show that a low content of sintering aids leads to insufficient liquid phase formation, hindered densification and porous defects inside the material, with spalling as the dominant RCF failure mode and the Weibull modulus being only 1.877. With the increase in sintering aid content, the liquid phase promotes densification and the growth of elongated β-Si_3_N_4_ grains; when the average grain aspect ratio reaches 4.47, the grain toughening mechanism significantly improves the RCF life, with the characteristic life attaining 1.035 × 10^7^ cycles. However, an excessive content of sintering aids induces the steric hindrance effect, which inhibits grain growth and increases the content of soft grain boundary phases, thus leading to the transition of the failure mode to wear and a subsequent decrease in service life. This study demonstrates that an appropriate liquid phase content is crucial for balancing the densification degree, grain morphology and RCF performance of Si_3_N_4_ ceramic bearing balls.

## 1. Introduction

Silicon nitride (Si_3_N_4_) ceramics are gradually replacing traditional metallic materials as the core rolling element material in all-ceramic or hybrid ceramic bearings for high-end equipment such as aerospace spindles, new energy vehicle electric drive systems, and precision CNC machine tools. This is due to their low density (approximately 40% of that of bearing steel), high elastic modulus, excellent chemical stability, and superior high-temperature mechanical properties. In particular, Si_3_N_4_ ceramic balls exhibit unmatched wear and fatigue resistance under extreme service conditions including high speed, lightweight design, and poor lubrication. However, the high-strength covalent bonds (Si-N bonds) inside the Si_3_N_4_ crystal lattice result in an extremely low atomic self-diffusion coefficient of the material, making it difficult to achieve full densification merely through solid-phase diffusion. Therefore, sintering aids are usually incorporated in industrial fabrication, and the full densification of Si_3_N_4_ ceramics is realized via the liquid-phase sintering mechanism. Among these, the Al_2_O_3_-Y_2_O_3_ system is selected in this study because it forms a low-melting eutectic phase that provides excellent wetting of Si_3_N_4_ particles and enables controlled liquid-phase sintering. This system is well-documented to promote both densification and the development of elongated β- Si_3_N_4_ grains, making it particularly suitable for systematically investigating the influence of sintering aid content on microstructure and rolling contact fatigue performance [1,2,3,4,5,6].

Based on the liquid-phase sintering theory proposed by Kingery [7,8,9,10], sintering aids react with the SiO_2_ oxide layer on the Si_3_N_4_ surface at high temperatures to form a low-melting-point eutectic liquid phase of oxides. This liquid phase not only acts as a lubricating medium for particle rearrangement, but also serves as a mass transfer channel to promote the dissolution–precipitation phase transformation of α-Si_3_N_4_ to β-Si_3_N_4_, thereby eliminating solid–gas interfaces and driving the shrinkage and densification of the sintered body. In this process, β-Si_3_N_4_ grains preferentially grow along the c-axis to form whisker-like elongated prismatic grains; this unique self-toughened microstructure endows Si_3_N_4_ ceramics with excellent fracture toughness through the mechanisms of crack deflection, crack bridging and grain pull-out.

Although the presence of a liquid phase is a prerequisite for achieving densification and microstructure regulation, the content and ratio of sintering aids exert complex dual effects on the final material properties. On one hand, studies by Rahaman et al. [11] confirm that increasing the liquid phase content within a certain threshold can significantly reduce the sintering activation energy and accelerate the densification kinetic process. Pyzik [12] further points out that a sufficient liquid phase environment is conducive to the development of β-phase grains, which directly determines the grain aspect ratio and the formation of interlocking grain structures. On the other hand, the glassy grain boundary phase remaining after sintering and cooling generally exhibits low strength and high brittleness. An excessively high content or inhomogeneous distribution of this phase can easily form stress concentration sources or softening points at high temperatures, thereby deteriorating the mechanical properties of the material.

Currently, numerous reports have been published on the correlation between sintering processes and the static mechanical properties of Si_3_N_4_ (e.g., bending strength, Vickers hardness) [13,14]. However, research on its rolling contact fatigue (RCF) behavior under dynamic alternating stress remains relatively scarce. RCF failure is a complex cumulative damage process involving subsurface crack initiation, propagation, and material spalling. It depends not only on the macroscopic hardness of the material but also on its microstructural characteristics (e.g., grain size, aspect ratio, content, and distribution of grain boundary phases). Existing studies have not yet systematically clarified how different sintering aid contents regulate microstructural parameters (e.g., α → β phase transformation rate, anisotropy of grain morphology, network topology of grain boundary phases) to further determine the evolution of RCF life and failure modes of ceramic balls.

In view of this, this study aims to establish a process-structure-fatigue property structure-activity relationship. Three types of Si_3_N_4_ ceramic bearing balls with gradient Al_2_O_3_-Y_2_O_3_ contents are prepared, and accelerated life tests are conducted under ultimate load conditions using a ball-on-plate contact fatigue testing machine. Combined with scanning electron microscopy (SEM), X-ray diffraction (XRD) and metallographic analysis technologies, the grain aspect ratio, distribution of grain boundary phases and phase composition under different sintering aid contents are systematically and quantitatively characterized. The intrinsic influence mechanisms of microstructural evolution on the RCF failure modes (the transition from spalling to wear) and life reliability are deeply discussed, which provides theoretical basis and experimental data support for the design of Si_3_N_4_ ceramic bearing balls with long service life and high reliability.

## 2. Materials and Methods

### 2.1. Sample Preparation

**Figure 1:**
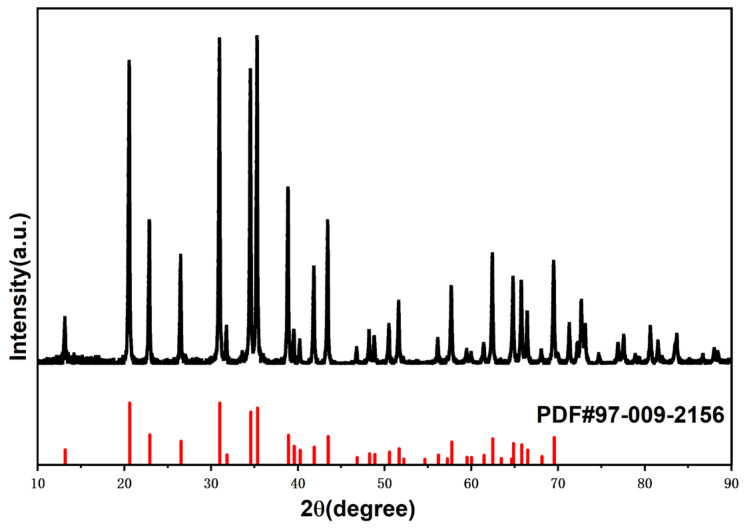
XRD of α-Si_3_N_4_ raw material.

The main components of the samples were α-Si_3_N_4_ powder (P-grade, α-Si_3_N_4_ ≥ 95 wt%, oxygen content ≤1 wt%, average particle size D_50_ = 0.7 μm, purity ≥ 99.9%, Vesta Si Co., Ltd., Ljungaverk, Sweden), Y_2_O_3_ powder (purity ≥99.9%, Sinopharm Chemical Reagent Co., Ltd., Shanghai, China), and Al_2_O_3_ powder (purity ≥ 99.9%, Zibo Xinfumeng Chemical Co., Ltd., Zibo, China). The XRD pattern of the α-Si_3_N_4_ powder is shown in Figure 1. The black one is the XRD pattern of the raw material, while the red one is the standard line of α-Si_3_N_4_ in PDF#97-009-2156. Three formulations with different sintering aid contents were designed (see Table 1), and the raw materials were mixed by planetary ball milling (φ800 × φ360 × 100, Zibo Rui’en Electronics Co., Ltd., Zibo, China) and then formed via cold isostatic pressing at 160 MPa for 600 s. The sintering process was implemented in two sequential steps: first, gas pressure sintering (GPS) was conducted at 1750 °C under a nitrogen atmosphere of 10 MPa with a holding time of 2 h; subsequently, hot isostatic pressing (HIP) treatment was performed at 1800 °C under 200 MPa for 4 h to eliminate residual pores in the samples. The final sintered compacts were subjected to rough grinding, fine grinding and precision polishing processes, and were fabricated into G5 precision ceramic balls with a nominal diameter of 9.525 mm. The material design and composition are presented in Table 1. At least five ceramic balls should be prepared for each component, and all these qualified samples are used for the subsequent rolling contact fatigue tests to ensure the reliability of the statistics (T30-60H, Luoyang Bearing Science and Technology Co., Ltd., Luoyang, China). Five balls were selected for testing in each group, and only the data of fatigue failure (spalling or excessive wear) occurring on the ceramic ball itself were recorded.

### 2.2. Performance Testing and Characterization

Bright and dark field observations are conducted using a metallographic microscope (SteREO Discovery.V20, ZEISS, Jena, Germany). The grain morphology and grain boundary phase distribution are analyzed via a field emission scanning electron microscope (FE-SEM, FEI Nova NanoSEM 450, Hillsboro, OR, USA). Both backscattered electron (BSE) mode and Compton backscattering (CBS) mode were employed to obtain phase contrast images. The CBS mode provides enhanced sensitivity to atomic number variations, enabling clearer differentiation between the Si_3_N_4_ matrix and grain boundary phases. For microstructural characterization, the ceramic balls were sectioned, polished using diamond suspensions down to 0.5 μm, and thermally etched at 1500 °C for 30 min to reveal grain boundaries. SEM imaging was performed at an accelerating voltage of 15 kV and a working distance of 10 mm. The grain aspect ratios are statistically measured using Image-Pro 6.0 software, with at least 300 grains measured per sample to ensure statistical significance. Both the mean values and standard deviations of the aspect ratios were calculated. The crystalline phase composition after sintering is analyzed with an X-ray diffractometer (XRD) (MiniFlex600, Rigaku, Tokyo, Japan). The XRD pattern of the original α-Si_3_N_4_ powder was also collected as a reference. Rolling contact fatigue tests were performed using a ball-on-disk contact fatigue tester (T30-60H, Luoyang Bearing Science and Technology Co., Ltd., Luoyang, China). And all the tests are conducted at room temperature and in a dry air environment, without any liquid lubrication. Failure criterion: spalling depth ≥ 0.05 mm or spalling area ≥ 0.5 mm^2^ for ball bearings. Rolling contact fatigue tests are performed on a ball-on-plate contact fatigue testing machine (T30-60H, Luoyang Bearing Science and Technology Co., Ltd., Luoyang, China). To accelerate the failure process, the rotational speed is set at 2500 r/min and the applied load at 5.5 kN (corresponding to a maximum Hertzian contact stress of approximately 6.8 GPa). Five balls were selected for testing in each group, and only the data of fatigue failure (spalling or excessive wear) occurring on the ceramic ball itself were recorded. The provided Weibull parameters are calculated based on the current sample size to obtain the confidence interval with statistical reliability.

## 3. Results and Discussion

### 3.1. Microstructure and Metallographic Analysis

**Figure 2:**
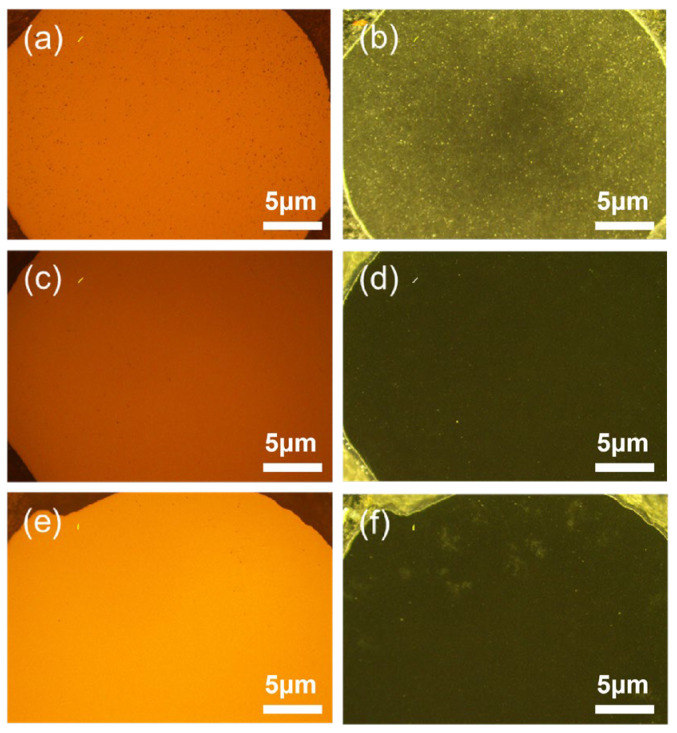
Metallographic micrographs of Si_3_N_4_ ceramics balls: Bright-field phase diagram (**a**) 4-1, (**c**) 4-2, (**e**) 4-3 and dark-field phase diagram (**b**) 4-1, (**d**) 4-2, (**f**) 4-3.

Figure 2 shows metallographic micrographs of polished cross-sections of the three Si_3_N_4_ ceramic ball samples. Based on the combined results of bright-field and dark-field observations, it can be concluded that there are no visible cracks, fractures, or inclusions on the surface of each sample, verifying the reliability of the pretreatment and forming-sintering process. The analysis of the microscopic morphological characteristics of samples with different additive contents is as follows: Sample 4-1 (low additive content, 8 wt%): Partial densification is incomplete, as shown in Figure 2a,b. The white spots observed in these micrographs correspond to residual surface pores, and the sample surface is covered with a large number of such fine pores. This is because the amount of sintering additive is low, and the volume fraction of the liquid phase generated at high temperatures is insufficient to completely fill the particle gaps or adequately wet the solid-phase particles. These residual pores act as stress concentration sites and play a critical role in the subsequent rolling contact fatigue failure behavior. According to the liquid-phase sintering theory, the lack of liquid phase significantly delays the diffusion and migration rates of substances, hinders the “dissolution–precipitation” process, and leads to a decrease in the sintering driving force, ultimately leaving residual pores that are difficult to eliminate within the material. Sample 4-2 (medium additive content): Uniform and dense structure. This sample presents a highly uniform surface morphology (Figure 2c,d), and no pores or defects are observed. This indicates that the 5 wt% additive formulation provides an appropriate amount of liquid phase at the sintering temperature, ensuring efficient mass transfer and densification contraction, while avoiding the organization segregation caused by excessive liquid phase, achieving an ideal microstructure. Sample 4-3 (high additive content): Component segregation and unevenness. Under dark-field illumination, sample 4-3 shows obvious irregular color difference areas on the surface (Figure 2f). This optical contrast inhomogeneity is mainly due to the introduction of excessive grain boundaries: On one hand, the locally enriched glass phase has differences in light scattering characteristics from the matrix; on the other hand, the high content of Y_2_O_3_ and Al_2_O_3_ is prone to solute atom segregation (Segregation) or non-uniform precipitation during the cooling crystallization process, resulting in non-homogeneous composition and distribution of the grain boundary phase.

### 3.2. Phase Composition Analysis

**Figure 3:**
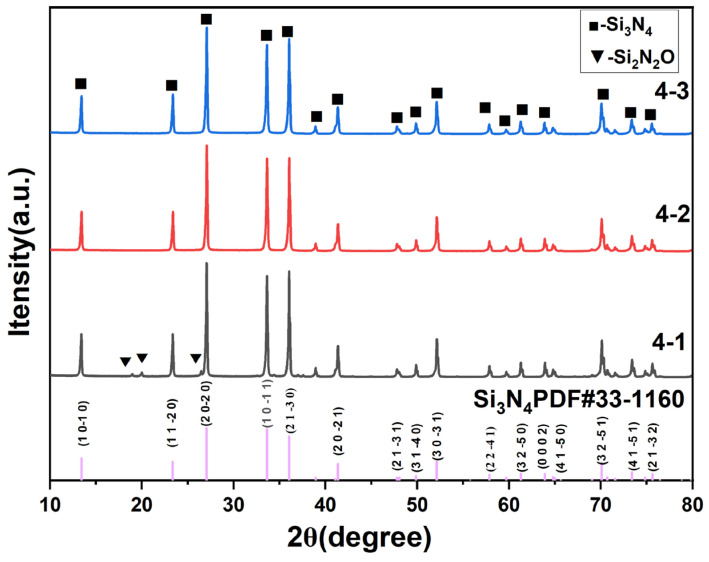
XRD phase analysis of Si_3_N_4_ ceramics.

Figure 3 shows the X-ray diffraction (XRD) patterns of Si_3_N_4_ ceramic balls with different sintering aid contents. The analysis results indicate that the main crystalline phase of all sintered samples is β-Si_3_N_4_, and no residual characteristic peaks of α-Si_3_N_4_ are detected. This confirms that under the current sintering process (1750 °C gas pressure sintering + 1800 °C hot isostatic pressing), the α → β phase transformation process proceeds completely, and the original powder achieves the reconstruction of phase structure through the dissolution–precipitation mechanism.

However, the composition of secondary phases shows significant differences among different samples. In Sample 4-1 with low sintering aid content, obvious diffraction peaks of Si_2_N_2_O crystalline phase appear in the 2θ ≈ 20–30° range of the XRD pattern. The mechanism of this phenomenon can be attributed to the imbalance of stoichiometric ratio of liquid phase components in the sintering system: when the addition amount of Y_2_O_3_-Al_2_O_3_ is low, it is insufficient to completely convert the natural oxide layer (mainly SiO_2_) on the powder surface into a liquid-phase glass network structure. According to thermodynamic equilibrium, the residual SiO_2_ reacts with the matrix Si_3_N_4_ in situ at high temperatures through the following reaction:

Si_3_N_4_ (s) + SiO_2_ (s) → 2Si_2_N_2_O (s)

The Si_2_N_2_O generated by this reaction usually exists at grain boundaries as a secondary phase, which may have an adverse effect on the fracture toughness of the material.

In contrast, no Si_2_N_2_O diffraction peaks are detected in the XRD patterns of Sample 4-2 and Sample 4-3. This indicates that with the increase in sintering aid content, sufficient metal oxides (Y_2_O_3_, Al_2_O_3_) effectively react with surface SiO_2_ to form a stable Y-Al-Si-O-N multi-component eutectic liquid phase. This liquid phase not only inhibits the formation of Si_2_N_2_O impurity phase, but also acts as a solvent to promote the densification process. After cooling, it is mainly stored at triple grain boundaries in the form of amorphous glass phase. No obvious Bragg diffraction peaks from this phase are observed in the XRD pattern (Figure 3) due to its amorphous characteristics, confirming its non-crystalline nature.

### 3.3. Evolution of Intergranular Phase and Grain Morphology

In this study, grain boundary phase refers to the amorphous phase that remains at grain boundaries and triple junctions after sintering and cooling. This phase originates from the liquid phase formed by the sintering aids during high-temperature processing. The measured grain boundary phase content in Table 2 represents the residual amorphous phase after sintering, quantified through image analysis of BSE-SEM micrographs.

**Figure 4:**
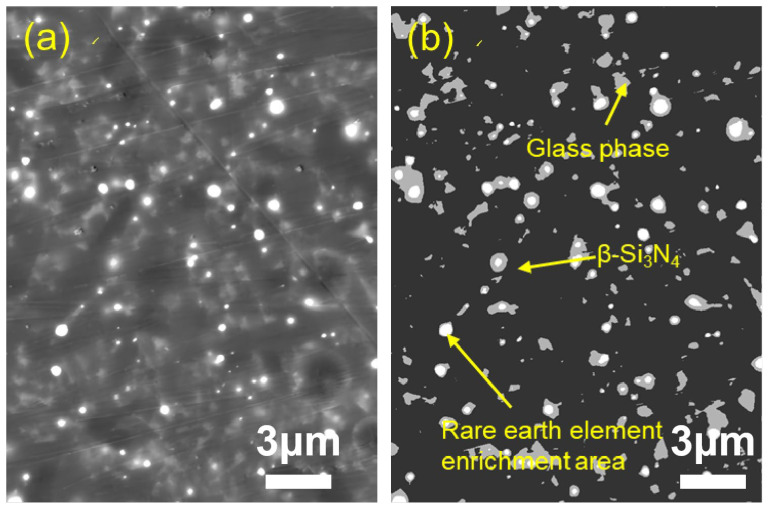
Morphological contrast of sample 4-1: (**a**) CBS image; (**b**) tricolor image.

**Figure 5:**
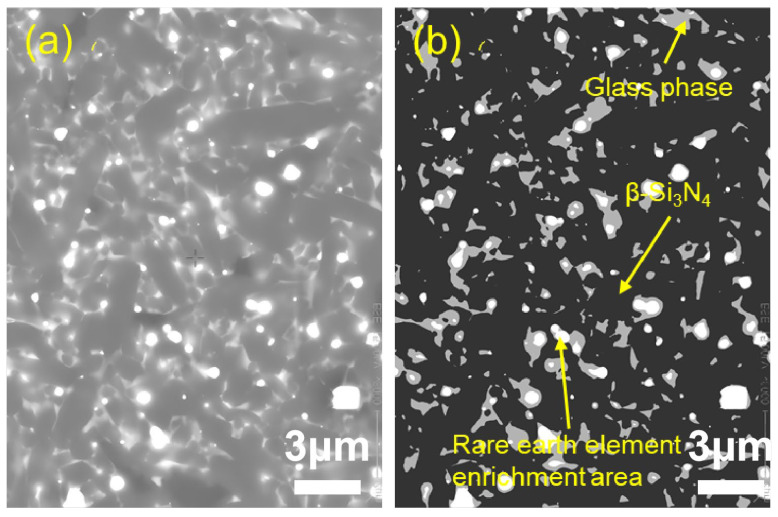
Morphological contrast of sample 4-2: (**a**) CBS image; (**b**) tricolor image.

**Figure 6:**
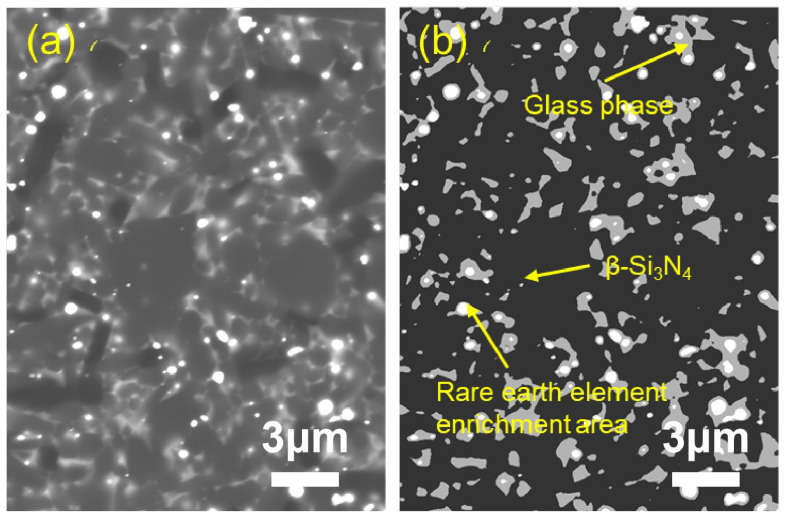
Morphological contrast of sample 4-3: (**a**) CBS image; (**b**) tricolor image.

To quantitatively characterize the distribution characteristics of the intergranular phase (IGP), the microstructure of the sample was observed using the backscattered electron (BSE) mode of the scanning electron microscope (Figure 4, Figure 5 and Figure 6). Since the imaging contrast of BSE mainly depends on the average atomic number (Z), a clear three-phase structure is presented in the figure: dark gray matrix β-Si_3_N_4_ grains (low Z value), white regions enriched with rare earth elements (high Z value), and light gray intergranular glass phase filling the gaps between the grains.

Table 2 shows the total amount of sintering additives added (as a percentage by mass). This refers to the initial content of Al_2_O_3_–Y_2_O_3_ added before sintering (as listed in Table 1). “The measured grain boundary phase (as a percentage by mass)” represents the residual amorphous phase content determined through quantitative analysis of backscattered electron (BSE) scanning electron microscope (SEM) images using gray threshold segmentation. These measured values are lower than the addition amount. The reasons for this include the volatilization of some sintering additives during the high-temperature treatment process, some cations being incorporated into the Si_3_N_4_ lattice, and the inherent limitations of two-dimensional image analysis in quantifying the three-dimensional phase distribution.

Quantitative statistics based on the gray threshold segmentation algorithm (Table 2) show that as the total content of sintering aids increases from 8 wt% to 12 wt%, the measured area fraction of residual grain boundary phases rises significantly from 7.6% to 11.7%. This indicates a positive correlation between the liquid phase content and the addition amount of sintering aids. It should be noted that the measured grain boundary phase content is consistently lower than the initially added sintering aid content, which can be attributed to partial volatilization of the sintering aids during the high-temperature sintering process, incorporation of some Y^3+^ and Al^3+^ ions into the Si_3_N_4_ crystal lattice, and the inherent limitations of two-dimensional image analysis in representing three-dimensional phase distributions. With the increase in sintering aids content, the liquid phase formed during sintering becomes more sufficient, which promotes better wetting, diffusion and homogeneous distribution of rare-earth elements. As a result, the rare-earth elements are more uniformly dispersed in the grain boundary phase rather than locally enriched, leading to a decrease in the number of obvious rare-earth enriched regions observed in Figure 5 and Figure 6. In Sample 4-3, the high-content grain boundary phases have transformed from the filling of isolated triple grain boundaries into a continuous network distribution, and this change in topological structure exerts a significant influence on the fracture behavior and high-temperature mechanical properties of the material.

### 3.4. The Dual Mechanisms of Grain Morphology Evolution

**Figure 7:**
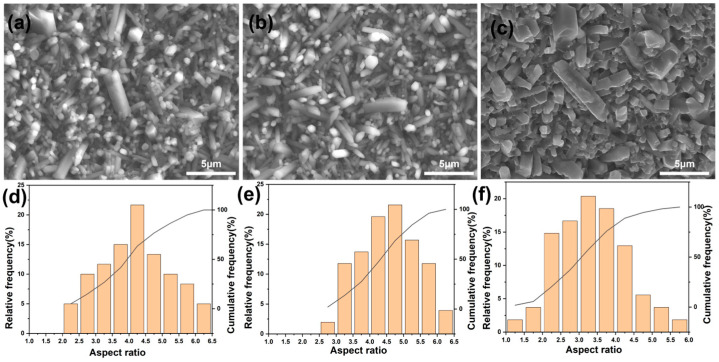
Scanning electron microscope and aspect ratio distribution of sintered samples: SEM image (**a**) 4-1, (**b**) 4-2, (**c**) 4-3; length-to-diameter ratio distribution graph (**d**) 4-1, (**e**) 4-2, (**f**) 4-3.

The grain aspect ratio (Aspect Ratio, AR) is a key microstructural parameter that determines the fracture toughness of Si_3_N_4_ ceramics. In the 5000-fold SEM image, the aspect ratios of 100 grains were statistically analyzed. The results are shown in Figure 7. The results reveal that the content of the sintering aid has a complex and non-monotonic influence on the growth kinetics of β-Si_3_N_4_ grains, presenting a dual effect of “first promoting and then inhibiting”: Anisotropic promotion of growth by the liquid phase (Sample 4-1 → 4-2): When the total sintering aid content increases from 8 wt% (4-1) to 10 wt% (4-2), the average aspect ratio of β-Si_3_N_4_ grains increases from 4.09 ± 0.52 to 4.47 ± 0.48. when the total sintering aid content is further increased to 12 wt% (4-3), the average aspect ratio drops sharply to 3.37 ± 0.41. The aspect ratio distributions for the three samples are shown in Figure 7d–f. This is because an appropriate low-viscosity liquid phase provides efficient channels for atomic diffusion, accelerating the migration of solute atoms (Si, N) from high-energy surfaces to low-energy surfaces (Ostwald ripening process). In an unrestricted environment, β-Si_3_N_4_ crystals tend to grow preferentially along the c-axis ([001] direction), developing into typical long columnar or needle-like morphologies, and this interlocked microstructure is conducive to improving the material’s toughness through the crack deflection mechanism. Spatial hindrance of excessive liquid phase and competing growth (Sample 4-2 → 4-3): However, when the aid content is further increased to excessive (4-3), the average aspect ratio drops sharply to 3.37. This anomaly is mainly attributed to the following mechanisms: Increased nucleation density: High concentrations of the liquid phase reduce the nucleation barrier, leading to a significant increase in the number of heterogeneous nucleation sites for the β phase. The simultaneous growth of a large number of nuclei triggers intense resource competition, limiting the final size of individual grains. Steric hindrance effect: As the grain density increases, the frequency of physical contact between grains rises, and the growth fronts of adjacent grains collide (Impingement), due to the lack of sufficient free space, the anisotropic growth along the c-axis of the grains is mechanically inhibited [15].Transformation of growth kinetics: An excessively thick grain boundary liquid phase film may change the anisotropy of interfacial energy, causing the growth rates in all directions of the crystal to tend to be consistent, thereby leading to a change in grain morphology from “elongated” to “short and thick”. Low sintering aid content leads to insufficient liquid phase, incomplete densification, residual porosity and low grain aspect ratio, which weaken the toughening mechanisms of crack deflection and bridging; optimal content realizes full densification and a peak grain aspect ratio to form an interlocked microstructure, thereby enhancing such toughening effects; excessive content causes a sharp drop in grain aspect ratio and accumulation of soft grain boundary glass phase, which impairs the toughening mechanisms. These microstructural changes and the corresponding variation in toughening ability show a strong correlation with the RCF spalling failure behavior of the samples: poor toughening induced by low/excessive contents accelerates crack initiation and propagation, leading to severe spalling failure, while the enhanced toughening from optimal content effectively inhibits crack extension and alleviates spalling.

### 3.5. Rolling Contact Fatigue (RCF) Behavior and Failure Mechanism

**Figure 8:**
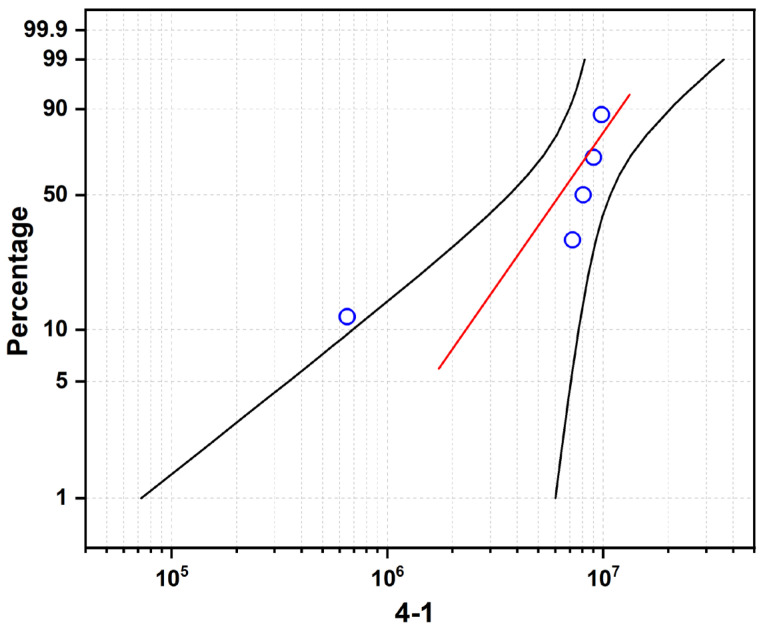
Rolling contact fatigue life curve of sample 4-1.

**Figure 9:**
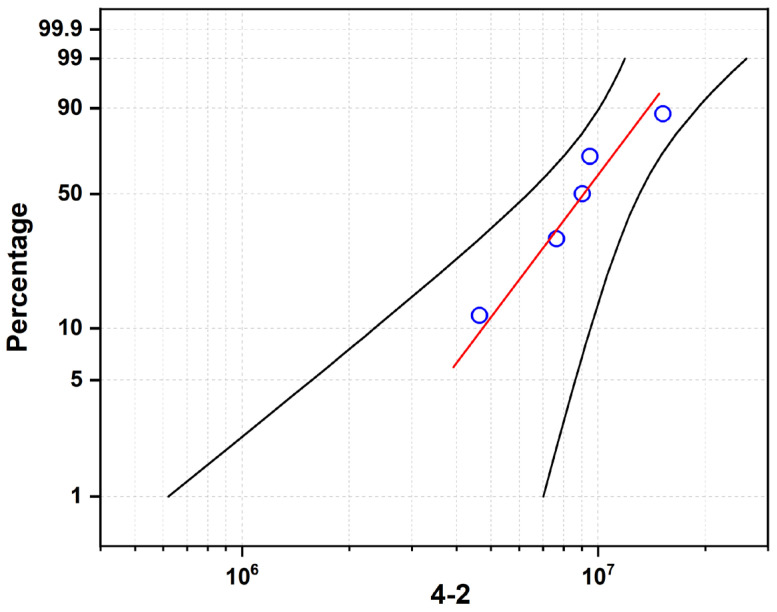
Rolling contact fatigue life curve of sample 4-2.

**Figure 10:**
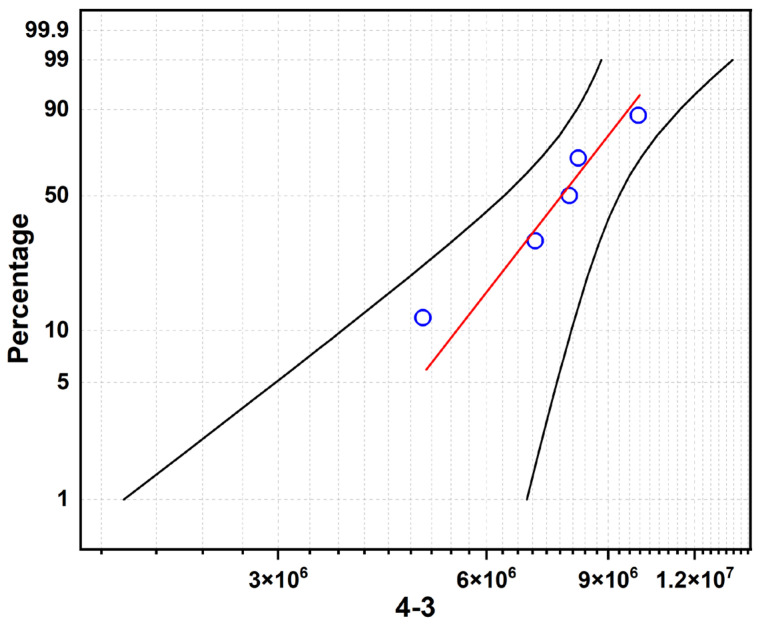
Rolling contact fatigue life curve of sample 4-3.

Figure 8, Figure 9 and Figure 10 present the Weibull probability plots for the three sample groups, where the blue circles represent the rolling contact fatigue life data of each Si_3_N_4_ ceramic bearing ball specimen. The red line is the theoretical fitting line calculated based on the Weibull distribution. The two black lines represent the upper and lower bounds of the confidence interval. These plots illustrate the variation in cumulative failure probability with increasing number of cycles to failure under a constant load of 5.5 kN.

For Sample 4-1 (Figure 8): The characteristic life (η) is 7.629 × 10^6^ cycles, and the Weibull modulus (m) is 1.877. The low m value indicates a wide scatter in fatigue life, which is attributed to the presence of residual pores and incomplete densification (as shown in Figure 2 and Figure 4). These defects act as random stress concentration sites, leading to inconsistent failure behavior.

For Sample 4-2 (Figure 10): This sample exhibits the highest characteristic life (η = 1.035 × 10^7^ cycles) with a moderate Weibull modulus (m = 2.872). The superior fatigue performance correlates with the high density and maximum grain aspect ratio (4.47) observed in this sample, where the interlocking grain structure effectively resists crack propagation.

For Sample 4-3 (Figure 10): The characteristic life decreases to 8.240 × 10^6^ cycles, while the Weibull modulus increases significantly to 5.389. The higher m value indicates improved reliability consistency, which can be attributed to the uniform distribution of the intergranular glass phase (Figure 6). However, the continuous network of soft grain boundary phase promotes wear-dominated failure, reducing the overall fatigue life compared to Sample 4-2.

Under the high-load acceleration test of 5.5 kN, the RCF lifetimes and failure modes of the three samples showed significant differences (Table 3, Table 4 and Table 5):

Sample 4-1 (spalling failure): Phenomenon: The main failure mode was surface spalling (Spalling). Mechanism: Due to the low density, the residual pores became a stress concentration source. Under the action of Hertz contact stress, cracks were initiated at the sub-surface maximum shear stress and extended to the surface, as confirmed by the fractographic evidence in Figure 8. Moreover, this sample had a relatively low grain aspect ratio, and the crack deflection and bridging toughening effect was weak, making the cracks prone to rapidly penetrate and form spalling pits. Lifespan: The characteristic lifespan was the lowest (7.629 × 10^6^ times), and the Weibull modulus was the lowest (1.877), showing a large degree of dispersion. The formation of Si_2_N_2_O secondary phase in sample 4-1 alters both the fracture toughness and RCF performance of Si_3_N_4_ ceramics. As a crystalline secondary phase, Si_2_N_2_O can induce crack deflection and consume fracture energy at grain boundaries, which may moderately improve fracture toughness, but it also disrupts the continuous intergranular glassy phase network and weakens the interfacial bonding between β-Si_3_N_4_ grains. Under repeated rolling contact stress, these weakened interfaces and residual Si_2_N_2_O particles act as preferential sites for microcrack nucleation and propagation, accelerating surface spalling failure and shortening the RCF life. Such behavior is consistent with previous studies, which confirm that excessive Si_2_N_2_O phase impairs the interfacial strength and fatigue resistance of Si_3_N_4_ ceramics [16].

Sample 4-2 (optimal performance): Phenomenon: It showed slight wear without early catastrophic spalling. Mechanism: The appropriate liquid phase ensured high density, while the highest grain aspect ratio (4.47) provided excellent fracture toughness, effectively inhibiting the expansion of fatigue cracks. Lifespan: The characteristic lifespan was the highest (1.035 × 10^7^ times), and the Weibull modulus was moderate (2.872) [14,17].

Sample 4-3 (grinding failure): Phenomenon: It occurred with a wide and deep wear (Wear), with a wear band width of 1276 μm. Mechanism: The continuous network of intergranular glass phase (visible in Figure 6) has a hardness significantly lower than that of β-Si_3_N_4_ grains, becoming preferential sites for wear under cyclic contact stress. At the same time, the decrease in grain aspect ratio weakened the overall toughness of the material. Lifespan: The characteristic lifespan decreased to 8.240 × 10^6^ times. However, thanks to the uniform distribution of the liquid phase, its Weibull modulus was the highest (5.389), indicating the best reliability consistency.

Wear in this work is the dominant RCF failure mode of the excessive sintering aid sample, caused by soft grain boundary glass phase acting as “soft points” under cyclic stress and undergoing preferential micro-fracture and detachment.

### 3.6. Comparison with State-of-the-Art Ceramic Bearing Materials

Our optimized Si_3_N_4_ ceramic balls with 10 wt% Al_2_O_3_–Y_2_O_3_ sintering aids achieved a characteristic RCF life of 1.035 × 10^7^ cycles under a maximum Hertzian contact stress of approximately 6.8 GPa. This performance compares favorably with recently reported results for Si_3_N_4_-based bearing materials. For instance, Liu et al. [1] investigated the rolling contact fatigue behavior of Si_3_N_4_ ceramic balls and reported characteristic lives in the range of 8.5 × 10^6^ to 9.2 × 10^6^ cycles under similar test conditions, demonstrating that our optimized composition yields superior fatigue performance. Additionally, recent studies on Si_3_N_4_ full ceramic bearings have demonstrated excellent tribological performance under self-lubrication conditions, with friction coefficients reaching as low as 0.000676 at 2800 N. Wang et al. further established a pseudo-dynamic model to calculate the allowable radial load for Si_3_N_4_ ceramic ball bearings to prevent secondary surface crack initiation, highlighting the importance of microstructural control in fatigue performance.

Compared to these recent studies, our work provides a systematic investigation of the relationship between sintering aid content, microstructure evolution (including quantitative analysis of grain aspect ratio and grain boundary phase distribution), and RCF failure modes. Specifically, we identify a non-monotonic relationship between sintering aid content and grain aspect ratio, and establish the synergistic toughening effect of “high density + high aspect ratio grains” as a core microstructure design principle for long-life Si_3_N_4_ ceramic balls. The identification of the optimal sintering aid composition (10 wt% Al_2_O_3_-Y_2_O_3_) and the elucidation of the transition in failure mode from spalling to wear with increasing additive content represent contributions that complement the existing literature by providing microstructural design guidelines specifically for RCF applications [18,19,20].

## 4. Conclusions

This paper systematically investigated the regulatory laws of Al_2_O_3_-Y_2_O_3_ sintering additives content on the microstructure evolution, phase composition, and rolling contact fatigue (RCF) behavior of Si_3_N_4_ ceramic bearings. By establishing the “process-structure-performance” relationship, the following main conclusions were drawn:

The non-monotonic regulatory effect of sintering additives on microstructure: The content of additives is the key factor determining the liquid phase sintering kinetics and grain morphology. At a low additive content (4% Al_2_O_3_ + 4% Y_2_O_3_), the lack of liquid phase leads to incomplete densification and residual porosity defects in the matrix and incompletely reacted Si_2_N_2_O phase; an appropriate amount of additives (5% Al_2_O_3_ + 5% Y_2_O_3_) provides the best mass transfer channels, promoting the preferential growth of β-Si_3_N_4_ along the c-axis, and achieving the peak average aspect ratio of grains (4.47); while excessive additives (6% Al_2_O_3_ + 6% Y_2_O_3_) trigger high-density nucleation and growth competition, and significantly inhibit the anisotropic growth of grains through steric hindrance effects, resulting in a reduction in the aspect ratio to 3.37 and a significant increase in the content of grain boundary glass phase to 11.7%.

The competition and transformation mechanism of RCF failure modes: This study reveals that the failure modes of Si_3_N_4_ ceramic balls under cyclic contact stress exhibit a transformation from “defect-induced spalling” to “microstructure-dominant wear”: In low-density samples, residual porosity acts as a significant stress concentration source, inducing the rapid initiation and expansion of subsurface cracks, leading to early catastrophic “spalling” failure. As the density increases, the failure dominant factor shifts to microstructure differences. In samples with excessive additives, the lower hardness of grain boundary glass phase cannot withstand the high Hertz contact stress (5.5 kN), and preferentially undergoes micro-region fragmentation and detachment, resulting in severe broadband wear.

The microstructure design principle for long-life ceramic balls: Sample 4-2 (5 wt% Al_2_O_3_ + 5 wt% Y_2_O_3_) exhibits the best rolling contact fatigue performance, with a characteristic life of up to 1.035 × 10^7^ cycles. Its excellent performance is attributed to the synergistic toughening effect of “high density + high aspect ratio grains”: The interlocking structure formed by long columnar grains effectively triggers crack deflection and bridging, significantly delaying the expansion rate of fatigue cracks.

It should be noted that this study has certain limitations: the RCF tests were carried out under fixed conditions (room temperature, dry air, no lubrication) with a single ball size (9.525 mm) and specific load (5.5 kN) and speed (2500 r/min). Future work will focus on investigating the RCF performance under diverse lubrication conditions, different service temperatures and various ball sizes to extend the research findings to practical engineering applications.

## Figures and Tables

**Table 1:** Experimental design table.

Sample	Sintering Aids (wt%)	Insulation Temperature (°C)	Insulation Time (min)
4-1	4%Al_2_O_3_ + 4%Y_2_O_3_	1750	120
4-2	5%Al_2_O_3_ + 5%Y_2_O_3_	1750	120
4-3	6%Al_2_O_3_ + 6%Y_2_O_3_	1750	120

**Table 2:** Microstructural parameters of Si_3_N_4_ ceramic bearing balls.

Sample	Grain Boundary Phase/wt%	Sintering Aids/wt%
4-1	7.6	1.7
4-2	8.5	3.1
4-3	11.7	2.3

**Table 3:** Stress cycle number of sample 4-1.

Sample	Si_3_N_4_ Balls Size/mm	Load Pressure/kN	Number of Cycles/10^7^	Is It Invalid?	Failure Mode
4-1	9.525	5.5	0.065	Failure	Spalling
4-1	9.525	5.5	0.982	Failure	Spalling
4-1	9.525	5.5	0.808	Failure	Wear
4-1	9.525	5.5	0.721	Failure	Wear
4-1	9.525	5.5	0.902	Failure	Wear

**Table 4:** Stress cycle number of sample 4-2.

Sample	Si_3_N_4_ Size/mm	Load Pressure/kN	Number of Cycles/10^7^	Is It Invalid?	Failure Mode
4-2	9.525	5.5	0.464	Failure	Wear
4-2	9.525	5.5	1.521	Failure	Wear
4-2	9.525	5.5	0.765	Failure	Wear
4-2	9.525	5.5	0.903	Failure	Wear
4-2	9.525	5.5	0.948	Failure	Wear

**Table 5:** Stress cycle number of sample 4-3.

Sample	Si_3_N_4_ Balls Size/mm	Load Pressure/kN	Number of Cycles/10^7^	Is It Invalid?	Failure Mode
4-3	9.525	5.5	0.486	Failure	Wear
4-3	9.525	5.5	0.791	Failure	Wear
4-3	9.525	5.5	0.814	Failure	Wear
4-3	9.525	5.5	0.995	Failure	Wear
4-3	9.525	5.5	0.706	Failure	Wear

## Data Availability

The original contributions presented in this study are included in the article. Further inquiries can be directed to the corresponding authors.
